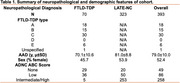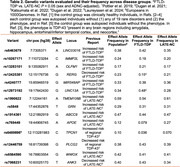# Genetic modifiers of TDP‐43 proteinopathy in FTLD‐TDP and LATE‐NC

**DOI:** 10.1002/alz70861_107942

**Published:** 2025-12-23

**Authors:** Seth D. Talyansky, Nadia Dehghani, Eddie B. Lee, David J. Irwin, David A. Wolk, Vivianna M Van Deerlin, Corey T. McMillan

**Affiliations:** ^1^ Department of Neurology, Perelman School of Medicine, University of Pennsylvania, Philadelphia, PA USA; ^2^ Department of Neurology, University of Pennsylvania Perelman School of Medicine, Philadelphia, PA USA; ^3^ Department of Pathology & Laboratory Medicine, Perelman School of Medicine, University of Pennsylvania, Philadelphia, PA USA

## Abstract

**Background:**

Transactive response DNA binding protein of 43 kDa (TDP‐43) aggregates are observed in cognitive disorders of aging including frontotemporal lobar degeneration (FTLD‐ TDP) and limbic‐predominant age‐related TDP‐43 encephalopathy neuropathologic change (LATE‐NC). Convergence and divergence in the genetic architecture of these disorders remains unclear. To further elucidate genetic modifiers of FTLD‐TDP versus LATE‐NC, we evaluated how genetic variants previously associated with TDP‐43 proteinopathy relate to neuropathological diagnosis.

**Methods:**

We identified 393 individuals in the Penn Integrated Neurodegenerative Disease Database with: (1) neuropathological diagnosis of FTLD‐TDP or LATE‐NC; (2) ABC score of Alzheimer’s disease neuropathologic change (ADNC); (3) SNP genotyping; (4) no pathogenic mutation (*GRN*, *C9orf72, etc*.); and (5) self‐reported White race (Table 1). We evaluated 14 genetic variants (MAF > 0.1, missingness < 20%) previously associated with autopsy‐confirmed FTLD‐TDP, LATE‐NC, or regional TDP‐43 pathology, comparing SNP frequency between the FTLD‐TDP and LATE‐NC groups using binomial logistic regression with additive models covarying for sex and ABC score (Table 2). We excluded FTLD‐TDP cases of type C or unspecified histological type in a sensitivity analysis.

**Result:**

Most LATE‐NC cases (78%) showed intermediate/high ADNC. Adjusting for sex and ADNC level, we observed higher frequency of rs12973192‐G (*UNC13A*, β = 0.61±0.25, *p* = 0.013) and lower frequency of rs12425381‐G (*RERG*, β = –0.68±0.30, *p* = 0.024) in FTLD‐TDP versus LATE‐NC at a nominal level of significance (*p* < 0.05). The remaining variants we studied did not differ in allele frequency between FTLD‐TDP and LATE‐NC after adjusting for sex and ADNC level. Excluding known and possible FTLD‐TDP type C cases strengthened the associations of rs12973192‐G and rs12425381‐G but did not yield new associations.

**Conclusion:**

A variant in *UNC13A* appears to increase risk of FTLD‐TDP relative to LATE‐NC, while a variant in *RERG* appears to decrease risk; this is directionally concordant with previously reported associations of these variants with FTLD‐TDP versus healthy controls. The potential implications of polygenic contributions to prognosis are an important area for future investigation.